# Mortality Related Risk Factors in High-Risk Pulmonary Embolism in the ICU

**DOI:** 10.1155/2016/2432808

**Published:** 2016-11-29

**Authors:** Begüm Ergan, Recai Ergün, Taner Çalışkan, Kutlay Aydın, Murat Emre Tokur, Yusuf Savran, Uğur Koca, Bilgin Cömert, Necati Gökmen

**Affiliations:** ^1^School of Medicine, Intensive Care Unit, Dokuz Eylül University, Izmir, Turkey; ^2^Dışkapı Yıldırım Beyazıt Education and Research Hospital, Medical Intensive Care Unit, Ankara, Turkey

## Abstract

*Introduction*. We sought to identify possible risk factors associated with mortality in patients with high-risk pulmonary embolism (PE) after intensive care unit (ICU) admission.* Patients and Methods*. PE patients, diagnosed with computer tomography pulmonary angiography, were included from two ICUs and were categorized into groups: group 1 high-risk patients and group 2 intermediate/low-risk patients.* Results*. Fifty-six patients were included. Of them, 41 (73.2%) were group 1 and 15 (26.7%) were group 2. When compared to group 2, need for vasopressor therapy (0 vs 68.3%; *p* < 0.001) and need for invasive mechanical ventilation (6.7 vs 36.6%; *p* = 0.043) were more frequent in group 1. The treatment of choice for group 1 was thrombolytic therapy in 29 (70.7%) and anticoagulation in 12 (29.3%) patients. ICU mortality for group 1 was 31.7% (*n* = 13). In multivariate logistic regression analysis, APACHE II score >18 (OR 42.47 95% CI 1.50–1201.1), invasive mechanical ventilation (OR 30.10 95% CI 1.96–463.31), and thrombolytic therapy (OR 0.03 95% CI 0.01–0.98) were found as independent predictors of mortality.* Conclusion*. In high-risk PE, admission APACHE II score and need for invasive mechanical ventilation may predict death in ICU. Thrombolytic therapy seems to be beneficial in these patients.

## 1. Introduction

Despite recent advances in prophylactic, diagnostic, and therapeutic modalities, pulmonary embolism (PE) is still one of the most important causes of hospital morbidity and mortality. Patients with PE have heterogeneous presentation and prognosis. While being treated PE has a short-term mortality of 1% in normotensive patients who do not have evidence of right ventricle (RV) dysfunction, the mortality rate rises up from 35 to 58% in patients with hypotension or shock [1–3]. Recently, early mortality risk assessment has become an important approach for optimal management of acute PE and the use of clinical models, for example, Pulmonary Embolism Severity Index (PESI), are suggested mainly for identifying low-risk PE [3, 4]. However, identification of intermediate- or high-risk patients with acute PE is more complicated and models which include clinical, laboratory, and imaging variables together are better in prediction of death [4–8]. In 2014, European Society of Cardiology (ESC) stratified the prognostic risk into three major categories: (i) high-risk group: hemodynamic unstable patients with RV dysfunction, (ii) intermediate-risk group: hemodynamic stable patients with either RV dysfunction or elevated biomarkers for cardiac injury (if both positive, intermediate-high; if either one or none positive, intermediate-low), and (iii) low-risk group [5].

High-risk PE, which was previously defined as massive PE, is relatively rare and accounts for less than 5% of all PE [9, 10]. Suspected high-risk PE is an immediate life-threatening situation. Most of these patients are usually admitted to intensive care unit (ICU) because of hemodynamic instability and severe hypoxemia or for thrombolytic therapy. However, there is limited data for mortality related factors during ICU stay. The primary outcome of this study was to identify possible risk factors associated with mortality in high-risk PE after ICU admission.

## 2. Patients and Methods

### 2.1. Study Population

The study was designed as retrospective cohort study and performed in ICUs of two reference hospitals between January 2012 and June 2016. After ethics committee approval, the database and medical records of both ICUs were screened for the admission diagnosis of PE. The need for informed consent was waived because of the study design. The inclusion criteria were as follows: (i) adult patients aged ≥ 18 years and (ii) confirmation of diagnosis with computerized tomography pulmonary angiography (CTPA). Patients who had a suspicion or diagnosis of PE with other modalities, such as ventilation/perfusion scan, were excluded.

### 2.2. Data Collection

Demographic data, comorbidities, clinical and laboratory data on admission, and acute physiology and chronic health evaluation (APACHE) II score were collected from the ICU database systems and each patient's medical records. Patients were screened for possible risk factors for PE. The risk factors were defined as follows: immobility, recent surgery within last month, recent travel within two weeks, cancer, congestive heart failure, chronic pulmonary disease, active smoking, obesity (body mass index > 30 m^2^/kg), oral contraceptive use in women of childbearing age or hormonal therapy in postmenopausal women, and previous venous thromboembolism history.

### 2.3. Radiologic Evaluation

All patients had CTPA within 24 hours after initial therapy/resuscitation and clinical stabilization. The localization of emboli was recorded. Central PE was defined as thrombus in the main pulmonary artery (PA) or right and left PA, whereas lobar thrombus was defined as any thrombus in lobar branches of pulmonary arteries. The diameters of main PA and left/right PA were recorded. If available, the results of lower extremity compression ultrasonography were recorded for the presence of acute and chronic deep venous thrombosis.

### 2.4. Echocardiographic Evaluation

Patients had echocardiographic evaluation either in the emergency room or soon after ICU admission. Data for RV dysfunction and systolic PA pressure and left ventricular ejection fraction (LVEF) were recorded. RV dysfunction was based on RV dilatation (end diastolic diameter > 30 mm) or hypokinesia or abnormal movement of the interventricular septum with or without tricuspid regurgitation [11].

### 2.5. Definitions for High-Risk PE

ESC criteria were used for risk stratification [5]. High-risk PE (group 1) was defined as acute PE with sustained systemic arterial hypotension with RV dysfunction. Intermediate and low-risk patients were classified into group 2: intermediate-risk PE was defined as the presence of RV dysfunction or cardiac injury confirmed by elevated cardiac enzymes in the absence of hypotension and PESI class >2. If none were present, patients were classified into low-risk group.

### 2.6. Therapies for PE

All patients had treatment for PE. The therapy was initiated with low molecular weight heparin (enoxaparin 1 mg/kg × 2 per day; subcutaneous). The use of enoxaparin was a standard approach in both ICUs because of incapacity of arrangement of heparin infusion and monitorization of activated partial thromboplastin time due to nurse staff shortage. Thrombolytic therapy was considered in hypotensive (systolic blood pressure < 90 mmHg) patients in the absence of any contraindications. The decision of thrombolytic therapy was made by a multidisciplinary team (pulmonologist, ICU physician, and cardiologist) in all patients according to current national and international guidelines. In patients who received thrombolytic therapy, medical records were screened for possible complications such as thrombocytopenia, gastrointestinal bleeding, cerebral hemorrhage, and hematomas.

All patients were treated with oxygen or mechanical ventilation support depending on severity of respiratory failure. Patients who presented with respiratory failure (respiratory rate > 35 breaths/min, paradoxical breathing pattern, and O_2_ saturation < 90% for more than 5 minutes) refractory to oxygen therapy and hemodynamic instability were intubated and invasively ventilated with standard ventilation protocols of each ICU. All other patients were treated with either oxygen therapy or noninvasive ventilation to keep oxygen saturation level >93–95% depending on patients status.

### 2.7. Statistical Analysis

We conducted a retrospective cohort study and reported its results in accordance with the STROBE (Strengthening the Reporting of Observational Studies in Epidemiology) guidelines [12]. The primary outcome of the study was to find out predictive factors related with ICU mortality in high-risk risk PE. All categorical variables are expressed as numbers and percentages and continuous variables were expressed as median and interquartile range (IQR). Categorical variables between groups were compared with chi-square or Fisher's exact tests; continuous variables were compared with Student's* t*-test or Mann–Whitney* U* test. The independent effect of each variable on mortality was assessed with multivariate logistic regression analysis backward conditional method. To build the model, a purposeful selection method was used to select a subset of covariates that were considered to be clinically important, adjusting for confounders and statistical significance. Because of the low number of the outcome variable (*n* = 13), we needed to select the most important clinical factors in the model. Age was not included into the model separately because of APACHE II score and need for vasopressor therapy was not included into the model because it was positive in all nonsurvivors. An adjusted odds ratio (OR) and a 95% confidence interval (CI) were reported for each independent factor. A two-tailed *p* value of <0.05 was considered statistically significant. Statistical analysis was performed with SPSS (Statistical Package for the Social Sciences Version 20; IBM Corporation, Armonk, NY, USA) program.

## 3. Results

**Figure 1:**
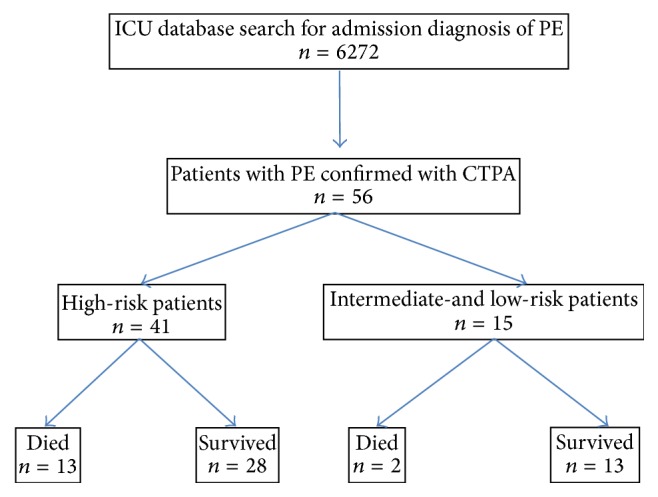
Study flowchart and ICU outcome of the study cohort (CTPA: computer tomography pulmonary angiography; ICU: intensive care unit; PE: pulmonary embolism).

A total of 56 patients (0.9% of all ICU admissions) were included into the study (Figure 1). Of them, 46.4% were male and the median age was 70.5 years (Table 1). The median APACHE II score was 18.0 (16.0–21.0). The most common comorbidities were hypertension (51.8%) and diabetes mellitus (26.8%). Risk factors (*n*) for PE were as follows: immobilization (41; 2 were after trauma), recent operation (22), cancer (9), congestive heart failure (8), previous venous thromboembolism (6), chronic respiratory disease (4), obesity (2), and recent travel (1).

### 3.1. Radiologic Findings

All patients had emboli in the main pulmonary vasculature and central PE was present in 46 (82.1%) patients (Table 1). Compression ultrasonography (*n* = 39) showed acute thrombi in 10 and chronic thrombi in 22 patients.

### 3.2. Echocardiographic Findings

Forty-five patients (80.4%) had RV dysfunction. The median PA pressure was 50 (45.0–60.0) mmHg. Median LVEF was 55.0 (55.0–60.0)%.

#### 3.2.1. Comparison of Group 1 and 2 Patients

Distribution of patients according to ESC criteria is presented in Table 2; 41 patients (73.2%) were classified into group 1, whereas 15 patients (26.8%) were classified into group 2 (Table 3). The percentage of central PE was similar between groups (85.4 versus 73.3%; *p* = 0.431). When compared to group 2, group 1 patients had statistically significant lower systolic (100.0 versus 90.0 mmHg; *p* < 0.001) and diastolic (68.0 versus 58.0 mmHg; *p* = 0.002) blood pressure; patients who had shock index > 1 were more common in group 1 (40 versus 78%; *p* = 0.011). Although the median PA pressure was higher in group 1, the difference did not reach a statistical significance (45.0 [40.0–55.0] versus 50.0 [45.0–60.0] mmHg; *p* = 0.406). Arterial blood gas analysis showed a higher pH level in group 2 than in group 1 (7.48 versus 7.43, *p* = 0.003); and arterial partial pressure for carbon dioxide did not differ between the groups. Partial pressure for oxygen was similar between groups. Nineteen patients (46.3%) in group 1 and 11 patients (73.3%) in group 2 were treated with oxygen therapy; seven patients (17.1%) in group 1 and 3 patients (20.0%) in group 2 were treated with noninvasive ventilation. Due to severity of respiratory failure, 15 (36.6%) patients in group 1 and 1 patient (6.7%) in group 2 were supported with invasive mechanical ventilation (*p* = 0.043). None of the patients needed vasopressor therapy in group 2, whereas 28 patients (68.3%) in group 1 needed vasopressors (*p* < 0.001).

#### 3.2.2. Thrombolytic Therapy

For thrombolysis, tissue plasminogen activator (tPA) was given with a dose of 100 IU in two hours in all patients. Thrombolytic therapy was administered to 29 patients in group 1. There was no statistically significant difference between the patients who were thrombolyzed and who had anticoagulation alone in group 1 (Table 4).

Thrombolytic therapy was administered in 4 patients in group 2 according to clinical decision. These patients were all intermediate-high-risk group.

Thrombolytic therapy related complications were observed in 3 patients (9.1%); two with nonserious bleeding at the injection sites and one hematoma formation in femoral area due to arterial cannulation.

#### 3.2.3. Mortality

Thirteen patients (31.7%) in group 1 and 2 patients (13.3%) in group 2 died during ICU stay (*p* = 0.306). Statistically significant factors associated with mortality in group 1 are presented in Table 5. There were more males in the nonsurvivors (32.1 versus 69.2%; *p* = 0.943). When compared to survivors, nonsurvivors had higher APACHE II score (18.0 versus 20.0; *p* = 0.002). More patients in the nonsurvivors needed vasopressor therapy (53.6 versus 100.0%; *p* = 0.003) and invasive mechanical ventilation (14.3 versus 84.6%; *p* < 0.001). The length of hospital stay was longer in survivors than nonsurvivors (15 and 5 days, resp.; *p* < 0.001).

In the unadjusted analysis, male gender (OR [95% CI] 4.75 [1.15–19.65]), APACHE II score > 18 (OR [95% CI] 13.75 [2.47–76.43]) and invasive mechanical ventilation (OR [95% CI] 33.00 [5.23–208.06]) were associated with increased risk of mortality (Table 6). In multivariate logistic regression analysis, APACHE II score > 18 (OR [95% CI] 42.47 [1.50–1201.1]; *p* = 0.028) and invasive mechanical ventilation (OR [95% CI] 30.10 [1.96–463.3] *p* = 0.015) and thrombolytic therapy (OR [95% CI] 0.03 [0.01–0.98]; *p* = 0.049) were found as independent predictors of mortality.

## 4. Discussion

In the present study, we wanted to assess the possible predictive factors for mortality in high-risk PE after ICU admission and found that APACHE II score > 18 and invasive mechanical ventilation increase the risk of death, whereas thrombolytic therapy has protective effect.

Hypoxemia (81%), increased alveolar-arterial gradient (80%), and hypocapnia (74%) are the most frequently observed gas exchange abnormalities seen in PE [13]. These abnormalities are associated with the size of the emboli, degree of obstruction, and the underlying cardiopulmonary disease. Severe forms of PE, because of either severe hypoxemia or shock related respiratory muscle insufficiency, may end up with a need for mechanical ventilation support. The reported incidence of respiratory failure is around 5% in massive PE [9]. However, we found a higher rate in the present study, the need of invasive mechanical ventilation was 36.6% in high-risk patients. Additionally, 17.1% of patients needed noninvasive ventilation. The cause of high rates of respiratory failure and invasive mechanical ventilation was probably because of the severity of these patients; most of the patients had shock index >1 and 68.3% needed vasopressor therapy. It was previously reported that the rate of respiratory failure could be as high as 47% in patients who need vasopressors [10].

We have found that invasive mechanical ventilation significantly increases the risk of death in high-risk PE. The negative effect of invasive mechanical ventilation was reported in some other studies as well [14–16] Soh et al. also reported that intubation was more frequent in the nonsurvivors in their study [14]. Khemasuwan et al. reported that the need for mechanical ventilation was associated with both ICU (OR, 12.0; 95% CI, 4.6–32.3) and hospital (OR, 11.9; 95% CI, 5.3–27.0) mortality [15, 16]. Positive pressure ventilation can have devastating effects on a patient with circulatory collapse from PE. Increases in intrathoracic pressure from positive pressure ventilation negatively affect cardiac output by decrease in venous return and preload [2]. In addition, induction agents used prior to intubation may have additive effect for decrease in blood pressure. Hypoxia and mechanical ventilation itself may increase pulmonary vascular resistance as well [13]. All these changes may result in hemodynamic collapse in PE. Most of these patients are currently ventilated with low positive end expiratory pressure (PEEP) and low tidal volumes to ensure low plateau pressure as recommended [2, 5, 13]. Mechanical ventilation is absolutely a life-saving procedure, but the optimal ventilation strategy for PE remains a challenge and more data are needed to understand the best mechanical ventilation approach. Although a group of patients in this cohort were supported by noninvasive ventilation, the efficacy and role of noninvasive ventilation in PE patients are yet another question waiting to be answered. Newer therapies like high flow oxygen therapy, extracorporeal membrane oxygenation (ECMO), and nitric oxide inhalation should be addressed in the future studies as well [2, 17].

APACHE II score > 18 was found as another independent predictor for mortality in this study. The cut-off value (>18) was chosen according the median scores of APACHE II of survivors and nonsurvivors. APACHE II score is generally accepted as a scoring system for critically ill patients, but its predictive yield for mortality in PE patients has been studied less. In one study, although the median APACHE II score was higher in nonsurvivors, it was not found as an independent predictor for mortality [18]. However, the study cohort consisted of moderately ill patients and was performed in a pulmonary clinic outside of ICU. Bach et al. compared the prognostic yield of different scoring systems and found that APACHE II score performed better than PESI and simplified PESI [19]. The good predictive yield of APACHE II in the critically ill depends on the global assessment of the patient. It consists of three parts: acute physiology parameters, comorbidities, and neurologic state (according to Glasgow coma scale score). These parameters are also known predictive factors for the outcome in PE. All these data suggest that APACHE II score could be used to predict death in PE patients admitted to ICU. We think that cut-off value of APACHE II score > 18 might be helpful for the identification of patients who have higher risk of death because of PE.

The recommended treatment of choice in high-risk PE is thrombolysis [5]. In this study, 70.7% of patients in group 1 received thrombolytic therapy, which is higher than that previously reported [9, 10]. Logistic regression analysis showed a beneficial effect of thrombolysis. This finding is similar to previously reported data [20]. There is good evidence on superiority of thrombolysis to heparin alone to accelerate lysis of emboli and restore hemodynamics and RV function [20, 21]. Most patients respond favorably to thrombolysis, as judged by clinical and echocardiographic improvement within 36 hours [22]. However, some patients deteriorate soon after onset of symptoms and die before ICU admission in massive PE [2]. It was previously shown that even delays in anticoagulation may cause increased risk of death in PE [14]. Therefore, timing of thrombolysis is crucial and prompt treatment is one of the most important factors for a better outcome in severe PE. An accelerated regimen of thrombolysis might be beneficial in critically ill PE patients [13, 23].

One of the important drawbacks of thrombolysis therapy is increased risk of bleeding. Overall rate for major bleeding is 10% [21]. In this study, although the thrombolysis rate is higher than previously reported, the complication rate was relatively low [9, 20]. We think that the main reason for low complication rate is multidisciplinary assessment for indications and possible contraindications for thrombolysis. However, it should also be noted that some occult bleedings might have been missed in the nonsurvivor group.

An interesting finding of this study was that male gender was associated with increased mortality; however, we were not be able to show an independent effect of gender in multivariate model analysis. High mortality rates in male patients were reported by some previous studies [24, 25]. A large database study reported that the overall crude 30-day mortality rate in PE was 8.9% for women and 9.8% for men and women had a lower risk of 30-day mortality in adjusted analysis (OR 0.8, 95% CI 0.7–0.9) [24]. Panigada et al. recently showed that although female PE patients were sicker and had echocardiographic signs of right heart dysfunction and positive troponins, mortality was lower in females; however, the difference did not reach a statistical significance [25]. The reason why males have higher risk of death remains unexplained; therefore, the effect of gender on PE associated mortality merits more research.

### 4.1. Strengths and Limitations

This study has several limitations. First, it is a retrospective study with limited number of patients, and the results cannot be generalizable. Second, in the study centers, serum brain-natriuretic peptide was not a routine evaluation laboratory test throughout the study period, and cardiac injury was assessed only by serum troponin levels. Third, none of the centers had therapeutic options other than thrombolysis such as surgical or catheter embolectomy, for this reason we were not be able to compare the effects of alternative therapy options. Last, we did not have detailed data for ventilatory settings (PEEP, tidal volume, and airway plateau pressure) in intubated patients which might also have an adverse effect on outcome.

On the other hand, we think that our study has some strengths. Our cohort consisted of very severe PE patients and the results of this study gives important insights to the most severe clinical presentation of PE. Literature data for this subgroup of patients are very limited and it has been shown that ICU admissions for PE showed a huge variation between hospitals; many patients are admitted to ICU with relatively weak indications [26]. Another strength is we have only included patients diagnosed by CTPA which is now considered as the main diagnostic method for the diagnosis of PE especially in the critically ill [5, 13].

## 5. Conclusion

Even with the recent improvements in diagnostic and therapeutic modalities, mortality for PE remains relatively high. The choice of treatment in PE depends on the estimated risk of poor outcome. Early identification of patients at risk is important not only to select the most appropriate treatment option but also to start it on time. In this regard, we think that APACHE II score > 18 and need for invasive mechanical ventilation may be helpful to intensive care physicians for further risk stratification in this fragile patient group. Urgent decision for other therapeutic options, like catheter/surgical intervention or ECMO, may be appropriate in severe patients who have either contraindication or limited response to thrombolysis. With respect to high mortality rates in intubated patients, we wonder whether a ventilation strategy specific for PE, as lung protective ventilation in acute respiratory distress syndrome, might be helpful. Further studies are needed to understand the best therapeutic approach and management strategy in critically ill PE patients.

## Figures and Tables

**Table 1:** Patient characteristics of study cohort (*n* = 56).

Age (years)	70.5 (61.3–77.8)
Male gender	26 (46.4)
Active smoking	4 (7.1)
APACHE II score	18.0 (16.0–21.0)
PESI score	133.5 (104.5–163.5)
*Comorbidities*	
Hypertension	29 (51.8)
Diabetes mellitus	15 (26.8)
Arrhythmia	10 (17.9)
Alzheimer's disease/dementia	9 (16.1)
Congestive heart failure	8 (14.3)
Coronary artery disease	8 (14.3)
Stroke	8 (14.3)
COPD	4 (7.1)
*Major symptoms on admission*	
Dyspnea	54 (96.4)
Pleuritic chest pain	33 (58.9)
Palpitation	28 (50.0)
Confusion	14 (25.0)
Syncope	12 (21.4)
Fever	10 (17.9)
Cough	10 (17.9)
Hemoptysis	3 (5.4)
*Electrocardiography findings*	
Sinus tachycardia	35 (62.5)
New RBBB	12 (21.4)
RV strain	25 (44.6)
S1Q3T3 pattern	19 (33.9)
*Echocardiography findings*	
PA pressure (mmHg)	50.0 (45.0–60.0)
RV dysfunction	45 (80.4)
LV ejection fraction (%)	55.0 (55.0–60.0)
*Computer tomography pulmonary angiography findings*	
Central PE	46 (82.1)
Lobar PE	10 (17.9)
Vessel diameter (mm)	
Main PA	29.8 (27.2–32.9)
Right PA	22.2 (20.1–26.1)
Left PA	23.7 (21.7–26.8)
Other findings	
Pleural effusion	21 (37.5)
Atelectasis	20 (35.7)
Ground glass appearance	16 (28.6)
Infarct	16 (28.6)
Reticular shadows	15 (26.8)
Consolidation	13 (23.2)
*Compression ultrasonography for lower extremities (available in 39 patients)*	
Acute thrombosis	10 (19.2)
Chronic thrombosis	22 (42.3)

All values are expressed as numbers (percentages) or median (interquartile range).

APACHE: acute physiology and chronic health evaluation; COPD: chronic obstructive pulmonary disease, ECG: electrocardiography; ICU: intensive care unit; LV: left ventricle; PA: pulmonary artery; PE: pulmonary embolism; PESI: pulmonary emboli severity index; RBBB: right bundle branch block; RV: right ventricle.

**Table 2:** Distribution of patients according to European Society of Cardiology risk class.

		Shock or hypotension	PESI classes III–V	RV dysfunction	Cardiac biomarkers
High-risk		+	+	+	+
*n* = 41					
Intermediate-risk	High	−	+	+	+
	*n* = 4				
	Low	−	+	Either one (or none) positive	
	*n* = 8				
Low-risk		−	−	Optional assessment	
*n* = 3					

PESI: pulmonary embolism severity index; RV: right ventricle.

**Table 3:** Admission clinical parameters of patients according to ESC classification (group 1: high-risk; group 2: intermediate- and low-risk).

	Group 1	Group 2	*p* value
	*n* = 41	*n* = 15	
Age (years)	72.0 (59.5–79.5)	68.0 (62.0–75.0)	0.493
Male gender	18 (43.9)	8 (53.3)	0.560
APACHE II score	18.0 (16.0–20.5)	18.0 (13.0–21.0)	0.243
Central PE in CTPA	35 (85.4)	11 (73.3)	0.431
Vital signs on admission			
Heart rate (beats/min)	111.0 (100–120)	105.0 (95.0–121.0)	0.572
Systolic blood pressure (mmHg)	90.0 (85.0–100.0)	110.0 (100.0–120.0)	<0.001
Diastolic blood pressure (mmHg)	58.0 (51.5–64.0)	68.0 (60.0–70.0)	0.002
Breathing frequency (/min)	22 (20–25)	22.0 (18.0–24.0)	0.675
Shock index > 1^*∗*^	32 (78.0)	6 (40.0)	0.011
PA pressure (mmHg)	50.0 (45.0–60.0)	45.0 (40.0–55.0)	0.406
Arterial blood gas on admission			
pH	7.43 (7.37–7.47)	7.48 (7.46–7.51)	0.003
PaCO_2_ (mmHg)	31.0 (27.2–35.9)	29.8 (23.7–35.5)	0.480
PaO_2_ (mmHg)	62.0 (48.0–81.7)	59.9 (47.6–67.2)	0.440
HCO_3_^−^ (mEq/L)	21.5 (18.3–23.5)	24.1 (19.5–26.2)	0.093
O_2_ saturation (%)	91.1 (84.2–97.0)	89.3 (81.3–94.0)	0.369
Lactate (mmol/L)	2.0 (1.1–3.3)	1.5 (1.1–2.1)	0.319
Troponin-T (ng/mL)	0.13 (0.05–0.73)	0.09 (0.05–0.47)	0.508
D-dimer (ng/mL)	7489 (2688–23476)	11000 (1807–16753)	0.627
Platelets (×10^3^/mcL)	223.0 (168.5–321.5)	264.0 (180.0–350.0)	0.566
Invasive mechanical ventilation	15 (36.6)	1 (6.7)	0.043
Need for vasopressor therapy^*∗∗*^	28 (68.3)	0 (0.0)	<0.001
Thrombolytic therapy	29 (70.7)	4 (26.7)	0.005
Mortality	13 (31.7)	2 (13.3)	0.306

All values are expressed as numbers (percentages) or median (interquartile range).

^*∗*^Shock index: heart rate/systolic blood pressure.

^*∗∗*^Vasopressors used for sustaining blood pressure were either noradrenaline or dopamine.

APACHE: acute physiology and chronic health evaluation; CTPA: computer tomography pulmonary angiography; PA: pulmonary artery; PaCO_2_: partial pressure of carbon dioxide; PaO_2_: partial pressure of oxygen; PE: pulmonary embolism.

**Table 4:** Comparison of group 1 patients according to therapy for pulmonary embolism.

	Thrombolysis	Anticoagulation	*p* value^*∗*^
	(*n* = 29)	(*n* = 12)	
Age (years)	70.0 (56.5–77.0)	73.5 (68.5–88.0)	0.094
Male gender	14 (48.3)	4 (33.3)	0.497
Cancer history	3 (10.3)	4 (33.3)	0.165
Cardiopulmonary resuscitation	11 (37.9)	5 (41.7)	1.000
APACHE II score	29.0 (17.0–20.5)	18.0 (15.0–23.0)	0.453
Central emboli on CTPA	24 (82.8)	11 (91.7)	0.423
PA pressure (mmHg)	50.0 (48.0–60.0)	47.5 (35.0–60.0)	0.287
Vital signs			
Heart rate (beats/min)	108.0 (98.5–120.0)	115.5 (107.0–120.0)	0.216
Systolic blood pressure	88.0 (83.5–97.0)	96.0 (88.5–102.0)	0.250
Diastolic blood pressure	58.0 (56.0–64.0)	56.0 (50.0–61.5)	0.328
Shock index >1^*∗*^	22 (75.9)	10 (83.3)	0.702
Cardiopulmonary resuscitation	16 (39.0)	0 (0.0)	0.003
Arterial blood gas analysis			
pH	7.43 (7.34–7.47)	7.43 (7.40–7.51)	0.158
PaCO_2_ (mmHg)	31.1 (27.3–36.6)	30.9 (26.5–33.7)	0.488
PaO_2_ (mmHg)	59.0 (47.3–78.1)	72.0 (57.1–92.7)	0.205
O_2_ saturation (%)	90.1 (79.8–95.6)	94.0 (91.4–97.3)	0.068
Troponin-T (ng/mL)	0.13 (0.03–0.71)	0.19 (0.08–1.00)	0.342
Invasive mechanical ventilation	11 (37.9)	4 (33.3)	0.536
Need for vasopressor therapy	19 (65.5)	9 (75.0)	0.719
Length of ICU stay (days)	4.0 (2.0–7.0)	3.5 (2.3–14.5)	0.944
Length of hospital stay (days)	12.0 (6.5–20.5)	14.0 (5.0–23.5)	*0.832*
ICU Mortality	8 (27.6)	5 (41.7)	*0.469*

All values are expressed as numbers (percentages) or median (Interquartile range).

APACHE: acute physiology and chronic health evaluation; CTPA: computer tomography pulmonary angiography; ICU: intensive care unit; PA: pulmonary artery; PaCO_2_: partial pressure for carbon dioxide; PaO_2_: partial pressure for oxygen.

^*∗*^Shock index: heart rate/systolic blood pressure.

**Table 5:** Statistically significant factors for mortality in group 1 patients (*n* = 41).

	Survivors	Nonsurvivors	*p* value
	(*n* = 28)	(*n* = 13)	
Male gender	9 (32.1)	9 (69.2)	0.043
APACHE II score	18.0 (16.0–20.0)	20.0 (19.0–30.5)	0.002
Cardiopulmonary resuscitation	3 (10.7)	13 (100.0)	<0.001
Invasive mechanical ventilation	4 (14.3)	11 (84.6)	<0.001
Need for vasopressor therapy	15 (53.6)	13 (100.0)	0.003
Hospital length of stay (days)	15.0 (10.0–24.8)	5.0 (4.5–8.0)	<0.001

All values are expressed as numbers (percentages) or median (interquartile range).

APACHE: acute physiology and chronic health evaluation.

**Table 6:** Odds ratios for mortality in group 1.

	Unadjusted odds ratio	95% CI	Adjusted odds ratio	95% CI
Male gender	4.75	1.15–19.65	16.67	0.79–350.00
APACHE II score > 18	13.75	2.47–76.43	42.47	1.50–1201.05
Invasive mechanical ventilation	33.00	5.23–208.06	30.10	1.96–463.31
Thrombolytic therapy	0.53	0.13–2.18	0.03	0.01–0.98

APACHE: acute physiology and chronic health evaluation; CI: confidence interval.
